# Untargeted Metabolomics Reveals Multiple Phytometabolites in the Agricultural Waste Materials and Medicinal Materials of *Codonopsis pilosula*

**DOI:** 10.3389/fpls.2021.814011

**Published:** 2022-01-10

**Authors:** Xu Zeng, Jiaxue Li, Xinkai Lyu, Juan Chen, Xiaomei Chen, Shunxing Guo

**Affiliations:** Institute of Medicinal Plant Development, Chinese Academy of Medical Sciences and Peking Union Medical College, Beijing, China

**Keywords:** untargeted metabolomics, plant waste, *Codonopsis pilosula*, lobetyolin, stems and leaves

## Abstract

*Codonopsis pilosula* has been used in traditional Chinese medicine for hundreds of years, where it has been used to treat anaemia, fatigue, a weak spleen, and stomach problems, among other ailments. The roots of *C. pilosula* are considered medicinal, while the aerial parts are always directly discarded after harvest in autumn or winter. Some studies have shown that the stems and leaves of *C. pilosula* also contain a variety of active metabolites, including saponins, flavonoids, terpenoids, and polysaccharides. To efficiently utilise resources, waste products from *C. pilosula* leaves and stems were analysed by untargeted metabolomics and chemometrics. A total of 1508 metabolites were detected and annotated, of which 463 were identified as differentially expressed metabolites (DEMs). These DEMs were grouped into classes, such as carboxylic acids and derivatives, steroids, organic oxygen compounds, fatty acyls, prenol lipids, and flavonoids. Metabolic profiling of *C. pilosula* tissues showed that the contents of polyacetylenes, polyenes, flavonoids, some alkaloids, steroids, terpenoids, and organic acids were higher in stems and leaves, whereas the contents of the main lignans and some alkaloids were more enriched in roots. Moreover, *C. pilosula* stems and leaves also contained a lobetyolin, syringin and atractylenolide III, which were detected by LC-MS/MS and HPLC-UV. The extracts of *C. pilosula* aerial parts also showed stronger antioxidant properties than roots. *C. pilosula* stems and leaves were rich in active ingredients and might have great value for development and utilisation.

## Introduction

*Codonopsis pilosula* (Franch.) Nannf. is a perennial herbaceous plant from the Campanulaceae family. This herb is mainly distributed throughout East, Southeast, and Central Asia (Gao et al., 2018). The dried roots of *C. pilosula* have been used as traditional Chinese medicine (TCM) for hundreds of years (Radix Codonopsis, “Dangshen” in Chinese, and “Tojin” in Japanese). This herbal medicine and its botanical product are widely used in clinical practice (Luan et al., 2021). Additionally, *C. pilosula* is also a traditional food and flavouring in Supplementary Materials. The National Health Commission of China published a list of substances that traditionally serve as both food and medicinal materials in 2020. *C. pilosula* is one of 9 model substances on that list and is typically used after it has been boiled, braised, or soaked in wine.

According to the Chinese Pharmacopoeia 2020 edition (China Pharmacopoeia Committee, 2020), the main herbal material of *C. pilosula* and more than 110 TCM preparations containing Radix Codonopsis are used for strengthening the spleen, tonifying the lung, nourishing the blood, and engendering liquid (Xie, 2008; Gao et al., 2018). Physiological functions have been widely reported (Chu et al., 2016; Ji et al., 2019; Bai R. B. et al., 2020; Gong et al., 2021), such as immunomodulatory, anti-viral, anti-fatigue, anti-hypoxia, antitumour, antioxidant, anti-inflammatory, neuroprotective, renoprotective, gastroprotective, and hepatoprotective effects. This herb has been clinically shown to treat hematopoietic dysfunction, coronary heart disease, hypotension, gastric ulcer, and chronic atrophic gastritis. As a healthcare product, it can also improve learning and memory abilities and delay senility. To date, more than 160 healthcare foods containing *C. pilosula* have been approved by China’s Food and Drug Administration. Therefore, *C. pilosula* is a major herbal material with a large market demand in East and South Asia. Moreover, approximately 200 phytometabolites have been isolated and identified from *C. pilosula* (Gao et al., 2018). The major bioactive components are a variety of polyyne and polyacetylene glycosides, polysaccharides, flavonoids, lignans, penoids, alkaloids, and lactones. Lobetyolin, a polyacetylene glycoside, has also been used to evaluate the quality of *C. pilosula* medicinal materials, as a standard component (China Pharmacopoeia Committee, 2020). Lobetyolin is also considered the major bioactive constituent in *C. pilosula*, reducing the ulcer index and gastrin content, significantly increasing the prostaglandin content, and elevating the epidermal growth factor to some extent (Gao et al., 2018; Bailly, 2021; Yoon and Cho, 2021).

The annual output of *C. pilosula* roots amounts to 5000 tons, whereas its aerial parts, i.e., the stems and leaves, are always directly discarded after harvest. This leads to resource waste, enormous economic loss, and serious environmental pollution. Therefore, methods of efficiently using *C. pilosula* stems and leaves are needed to generate value as food or health products. Previous study has shown that whole plants of *C. pilosula* contain various bioactive substances and abundant nutritional components (Chen M. et al., 2020). However, comprehensive chemical composition analysis has not been performed on the aerial parts (withered stems and leaves) and medicinal materials (dried roots) of *C. pilosula* after harvest. In our study, untargeted metabolome comparisons were performed on these two groups of plant parts. Lobetyolin was also detected by quantitative analysis in *C. pilosula* stems and leaves. Our data revealed the active compounds and therapeutic healthcare value from the agricultural waste materials of *C. pilosula*.

## Materials and Methods

### Standards and Reagents

Methanol, acetonitrile, and chloroform (HPLC grade) were purchased from Fisher Chemical Co., Inc. (Geel, Belgium). Lobetyolin (reference standard, CAS No. 136085-37-5), syringin (CAS No. 118-34-3) and atractylenolide III (CAS No. 73030-7-14) was purchased from Yuanye Bio-Technology Co., Ltd. (Shanghai, China). All other chemicals and solvents were analytical grade and purchased from common sources.

### Samples

**FIGURE 1:**
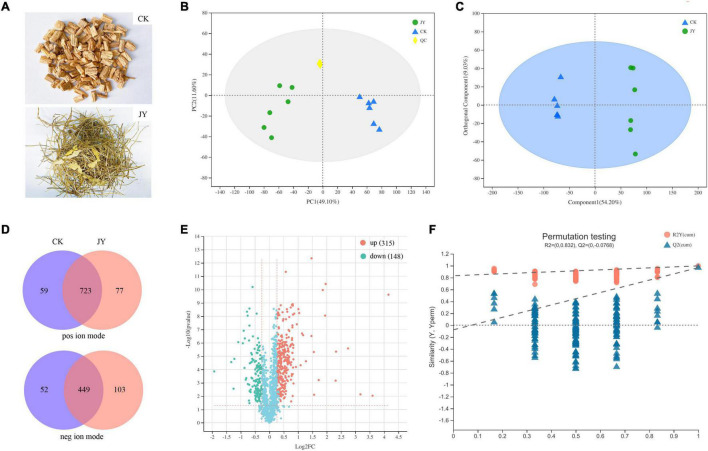
Overview of metabolomics in *Codonopsis pilosula*. Samples **(A)**, principal component analysis (PCA) **(B)**, orthogonal partial least squares discriminate analysis (OPLS-DA) **(C)**, score plots Venn diagram **(D)**, volcano plot **(E)** and OPLS-DA model permutation test **(F)** of metabolites for comparison between JY and CK group.

In autumn, *C. pilosula* roots, stems, and leaves were collected from a Chinese herbal medicine plantation at Shanxi Zhendong Pharmaceutical Co., Ltd. (Changzhi, China). After washing, all samples were dried and crushed to a coarse powder. Then, two groups, the aerial parts (withered stems and leaves, JY group) and medicinal materials (dried roots, CK group) of *C. pilosula*, were prepared (Figure 1A).

### Metabolomics Analysis

For *C. pilosula* aerial parts and roots, six biological replicates from each group were assayed for metabolomics analysis. Based on previous reports (Xu et al., 2020; Ma et al., 2021), metabolite extraction and UPLC-MS were performed. In brief, 50 mg of sample was precisely weighed, followed by ultrasonic extraction with a methanol buffer (0.4 mL, 80% aqueous) and 20 μL of internal standard (0.3 mg/ml 2-chloro-D-phenylalanine) for 30 min and incubated for 30 min at −20°C. The 200 μL supernatant was transferred into a new vial for UPLC-MS analysis after centrifuging at 13000 × *g* for 15 min at 4°C. Quality control (QC) samples were prepared by combining 10 μL supernatant from each test sample. To monitor the stability and repeatability, QC samples were injected every six test samples throughout the analytical run.

LC-MS was performed on a Waters UPLC system (Waters, Milford, CT, United States) coupled with a Q Exactive HF-X Mass Spectrometer (Thermo Fisher Scientific, San Jose, CA, United States). LC separation was performed using an Acquity BEH C18 column (100 mm × 2.1 mm i.d., 1.7 μm; Waters, Milford, CT, United States). Solvent A was water containing 0.1% (v/v) formic acid, and solvent B was acetonitrile containing 0.1% (v/v) formic acid. During separation, the flow rate was 0.40 mL/min, the column temperature was set at 40°C, and the injection volume was 2.00 μL. The solvent gradient was as follows: 0-24.5% B over 0-3.5 min, 24.5-65% B over 3.5-5 min, 65-100% B over 5-5.5 min, 100% B over 5.5-7.4 min, 100-51.5% B over 7.4-7.6 min, and 51.5-0% B over 7.6-7.8 min, and maintained at 0% B until 10 min. The MS data were collected using a mass spectrometer equipped with an electrospray ionisation (ESI) source operating in either positive or negative ion mode. The parameters of mass spectrometry were set as follows: spray voltage (+), 3500 V; spray voltage (−), 3500 V; capillary temperature, 325°C; sheath gas, 50 arb; auxiliary gas, 13 arb; probe heater temperature, 425°C; normalised collision energy (NCE), 20-40-60 V; and mass spectrum scanning range, 70–1050 m/z.

According to the description in previous studies (Xu et al., 2020; Ma et al., 2021), the raw data were imported into the Progenesis QI 2.3 (Waters Corporation, Milford, CT, United States) for baseline filtration, peak identification, integration, retention time correction, peak alignment, and uniformisation. The pre-processing results generated a data matrix containing the retention time (RT), mass-to-charge ratio (m/z), and peak intensity. SIMCA-P + 14.0 was used to perform clustering by principal component analysis (PCA) and orthogonal partial least squares discriminate analysis (OPLS-DA) methods. OPLS-DA was performed to determine global metabolic changes between comparable groups. Statistically significant differences between groups were selected with variable influence on projection VIP > 1, *p* < 0.05, and fold change > 1.2 or < 0.833. Metabolites were summarised, annotated, and analysed for their physical and chemical properties and biological functions based on public databases, namely METLIN^1^, Human Metabolome^2^, and KEGG databases (using MetaboAnalyst^3^). For phytochemical compounds, more than 230 metabolites identified in *C. pilosula* from previous studies were also analysed separately in our data (Liu et al., 2003; Gao et al., 2015; Chen et al., 2018; Gao et al., 2018).

### Determination of Lobetyolin by LC-MS/MS

Referring to the method recorded in the China Pharmacopoeia Committee (2020) and previous study (Dong et al., 2021), dried aerial parts of *C. pilosula* were used for lobetyolin extraction by the ultrasound-assisted method. Briefly, 1 g powder was transferred to a conical flask with 50 mL of methanol. Extraction proceeded for 30 min at 100 W and 40 kHz. After 10 min, the supernatant fluid was further diluted 2500-fold and filtered through a 0.22-μm membrane before injection into the UPLC-MS/MS system for analysis.

The reference standard lobetyolin was applied to identify the chromatography peaks. The standard chemicals were accurately weighed and dissolved in methanol to a concentration of 1 mg/ml. Stock solutions with final concentrations of 0.1 mg/ml were prepared in methanol, and the standard dilutions were 0.5, 1, 5, 10, 50, 100, and 200 ng/mL.

The HPLC system consisted of an Agilent 1290-6460 LC–MS/MS instrument (Agilent, United States). Chromatographic separation was achieved using an Agilent C18 (2.1 mm × 100 mm, 3 μm) column at 35°C, and the flow rate was 0.3 mL/min. Then, 5 μl injections were gradient eluted using a mobile phase consisting of (A) water (containing 0.1% formic acid) and (B) methanol with the following gradient procedure: 0 min, 40% B; 0–1 min, 40–60% B; 1–3 min, 60–90% B; 3–4 min, 90% B; and 4.01–6 min, 40% B. Lobetyolin was achieved in negative ESI mode. The column effluent was monitored by Agilent 6460 triple quadrupole LC/MS using dynamic Multiple Reaction Monitoring (MRM). The parameters were optimised and listed as follows: ion spray voltage, 5500 V; collision energy, 30 V; and capillary temperature, 550°C. The LC-MS/MS analysis of lobetyolin produced a negative ion of m/z 441.4, corresponding to the [M + COOH]^–^ precursor ion. Likewise, the mass transition patterns m/z 441.4 + 179.0 and 441.4 + 185.4 were selected for the identification and quantification of lobetyolin. Between-group comparisons were analysed by Student’s *t*-test for unpaired data using SPSS 19.0. Differences at *p* ≤ 0.05 were considered significant.

### Determination of Syringin and Atractylenolide III by HPLC

Referring to the previous study (Bai Y. E. et al., 2020), the aerial parts and roots of *C. pilosula* were used for syringin and atractylenolide III extraction by the ultrasound-assisted method. Briefly, 1 g powder was extracted with 25 mL of 75% methanol in an ultrasonic bath for 45 min. The solution was dried in a water bath and redissolved in 5 ml of methanol. The solution was filtered through a 0.45-μm membrane before injection into the HPLC system for analysis. The standard dilutions were 1, 10, 20, 50, 100, and 200 μg/mL for syringin and atractylenolide III.

The HPLC system: a Waters 600 controller pump, a Waters 2707 auto-sampler, a Waters 2996 detector (Waters Co., Milford, MA, United States). HPLC chromatography was adopted with as the chromatographic column Agela venusil ASB-C18 (4.6 mm × 250 mm, 5 μm) at room temperature and a gradient elution compose of 0.1% phosphoric acid solution (phase A) and acetonitrile (phase B) at flow rate of 0.8 mL. The detection wavelength was 220 nm. The linear gradient elution program: 5–10% B at 0–10 min; 10–15% B at 10–30 min; 15–20% B at 30–50 min; 20–30% B at 50–65 min; 30–50% B at 65–75 min; 50–70% B at 75–80 min; 70% B at 80–85 min; 70–80% B at 85–90 min; 80–100% B at 90–95 min; 100% B at 95–120 min. Data processing was performed using Empower software (Waters, version 5.00). Between-group comparisons were analysed by Student’s *t*-test for unpaired data using SPSS 19.0. Differences at *p* ≤ 0.05 were considered significant.

### Antioxidant Activity

Referring to a previous study (Tang and Chen, 2021), 1 g powders of *C. pilosula* dried aerial parts and roots were extracted with 25 mL of 75% methanol in an ultrasonic bath for 45 min. The solution was dried in a water bath and redissolved in 5 ml of water. Ferric-reducing antioxidant power (FRAP) assays and ABTS radical scavenging assays were performed according to the description in kit purchased from Nanjing Jiancheng Bioengineering Institute. The absorbance values were detected at 405 nm and 593 nm, respectively. All measurements were done in triplicate.

## Results and Discussion

### Overview of Metabolomics

While roots are the main herbal material from *C. pilosula*, multiple active metabolites were also produced in other tissues. An untargeted metabolomics study on the various tissues of *C. pilosula*, i.e., the roots (CK group) and the stems and leaves (JY group), with six biological replicates was carried out using the protocol with UPLC-MS and an integrated informatics pipeline. After removing redundancy and noise signals, which are indicative of poor quality or non-biological origin, 10731 and 5994 spectral signals were identified from positive and negative ion modes, respectively (Supplementary Table 1). Metabolite annotation was performed by comparison of the accurate m/z-values. In total, 1508 metabolites were annotated in *C. pilosula* samples using our integrated bioinformatics pipeline (Supplementary Table 2).

The base peak intensity chromatograms of QC samples and standard curve are shown in Supplementary Figures 1, 2. As shown in PCA score plots (Figure 1B), QC samples were clustered together, suggesting that our method had good stability and reproducibility. Unlike the PCA model, previous studies indicated that the OPLS-DA model could reduce system noise and extract variable information. As shown in Figure 1C, the OPLS-DA scores plot for the two groups had strong clustering, without any overlap. In the permutation test (Figure 1F), the R^2^X, R^2^Y (goodness-of-fit parameter), and Q^2^ (predictive ability parameter) of the OPLS-DA model were 0.617, 0.983, and 0.969, respectively, indicating the repeatability of the model.

In our study, 723 and 449 metabolites were annotated and identified in both groups from positive and negative ion modes, respectively (Figure 1D). In the comparison of the JY and CK groups, the significantly differentially expressed metabolites (DEMs) were separated based on the criteria VIP > 1, *p* < 0.05 and fold change > 1.20 or < 0.833. Taken together, 315 and 148 annotated metabolites (Figure 1E) were detected as up- and down-regulated metabolites, respectively.

**FIGURE 2:**
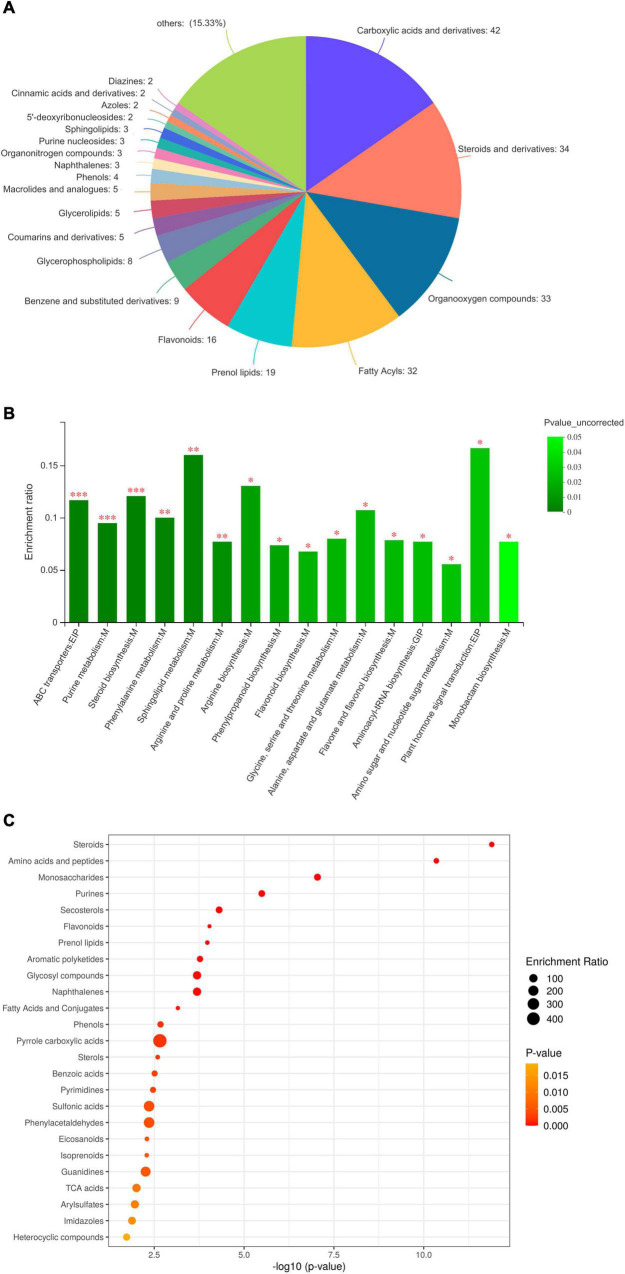
Functional annotation and classification of differential metabolites identified in the comparison between JY and CK group. **(A)** Classification of differentially expressed metabolites (DEMs), **(B)** KEGG enrichment analysis of DEMs, and **(C)** Bubble plot of KEGG pathway of DEMs.

In total, 463 DEMs were categorised into 20 classes based on the HMDB database (Figure 2A). DEMs were grouped into classes of carboxylic acids and derivatives (42 metabolites), steroids (34 metabolites), organic oxygen compounds (33 metabolites), fatty acyls (32 metabolites), prenol lipids (19 metabolites), flavonoids (16 metabolites), and others, such as benzene, glycerophospholipids, and coumarins. Based on the KEGG database (Figures 2B,C), most DEMs were enriched in steroids, amino acids and peptides, saccharides, purines, flavonoids, prenol lipids, aromatic polyketides, glycosyl compounds, fatty acids, and phenols. Therefore, the untargeted metabolomics detected various metabolites of steroids, organooxygen compounds, fatty acyls, flavonoids, glycerophospholipids, and aromatic polyketides, among others, in *C. pilosula* stems and leaves. Moreover, to understand the metabolic activities in *C. pilosula* tissues, we mapped the DEMs to KEGG pathways (Figure 2C). In comparing *C. pilosula* from the JY group to that from the CK group, most DEMs were involved in “flavonoid biosynthesis,” “betalain biosynthesis,” “anthocyanin biosynthesis,” “steroid biosynthesis,” “isoquinoline alkaloid biosynthesis,” “phenypropanoid biosynthesis,” “purine metabolism,” “sphingolipid metabolism,” and “amino acid metabolism,” such as phenylalanine, arginine, proline, and alanine. In summary, DEMs clustering of JY samples against the CK sample suggested that *C. pilosula* stems and leaves could also contain multiple active ingredients.

### Overview of Phytometabolites

According to previous studies, more than 230 phytometabolites from *Codonopsis* have been reported (Gao et al., 2018), including 23 polyacetylenes and polyenes, 21 flavonoids, 2 coumarins, 20 lignans, 29 alkaloids, 48 terpenoids, 15 steroids, 28 organic acids, and 45 other metabolites. Here, most of them were identified in the samples from the JY or CK groups (Supplementary Table 3). In total, 22 polyacetylenes or polyenes, 18 flavonoids, 2 coumarins, 13 lignans, 26 alkaloids, 14 terpenoids, 9 steroids, 27 organic acids, and 34 other metabolites were detected in positive or negative mode, respectively. The DEMs between the JY and CK groups included lobetyol, apigenin, luteolin-glucopyranoside, cynaroside, tangshenoside, codotubulosine, codonopiloside, codonopsine, oleanolic acid, stigmasterol, caffeic acid. However, there were no differences in some functional ingredients between the two groups, such as lobetyolin, codonopilodiynoside A, and quercetin.

### Polyacetylenes, Polyenes, and Their Glycosides

Polyacetylenes, polyenes, and their glycosides are widely distributed in the medicinal plants of *Codonopsis*. As shown in Supplementary Table 3, 9 of 22 polyacetylenes and polyenes were identified as DEMs in the comparison of the JY and CK groups (Table 1). Compared with the roots of *C. pilosula*, most were upregulated in the stems and leaves, including lobetyol, pilosulyne, and codonopilodiynoside D. Lobetyol, one of the active components in *C. pilosula*, has been reported to possess specific bioactive properties, such as anti-virus, anti-inflammation, and proliferation inhibition functions (Shen et al., 2016). In 2015, pilosulynes A–G, which are five polyynes and two polyenes, were first isolated from *C. pilosula* roots. Among them, pilosulyne F exhibited anti-HCV (hepatitis C virus) activity (Liao et al., 2015). However, lobetyolin (the standard chemical marker for Radix Codonopsis in Chinese Pharmacopoeia) and lobetyolinin, the mono- and bis-glucosylated forms of the polyacetylenic compound lobetyol, were not identified as DEMs in metabolomics analyses (He et al., 2020). Many studies have shown that lobetyolin and lobetyolinin have multiple pharmacological effects, including anti-cancer, anti-inflammation, antioxidative, and xanthine oxidase-inhibiting properties (Bailly, 2021). Overall, our results implied that most of the DEMs identified as polyacetylenes and polyenes accumulated more in the stems and leaves of *C. pilosula*.

**TABLE 1:** Differentially expressed metabolites (DEMs) annotated as polyacetylenes, polyenes and their glycosides.

Polyacetylenes and polyenes	Exact mass	Formula	ID_[M + H] +	Fold_change	ID_[M-H]-	Fold_change
lobetyol	234.1256	C14H18O3	metab_599/metab_2560/ metab_2797/metab_5068/ metab_6240/metab_10051/ metab_12673	metab_2797: 1.29	metab_22726/metab_16790	
Pilosulyne A	252.1361	C14H20O4	metab_5853	metab_5853: 1.30	metab_18580/metab_18105	
Pilosulyne C	236.1412	C14H20O3	metab_204/metab_342/ metab_6465	metab_204: 1.24; metab_342: 1.25;	metab_22708/metab_22909	
Pilosulyne F	254.1518	C14H22O4	metab_2670/metab_5383	metab_2670: 1.56;	metab_19148/metab_18513	
Pilosulyne G	252.1361	C14H20O4	metab_5853	metab_5853: 1.30;		
9-(tetrahydropyran-2-yl)-non-trans-8-ene-4,6-yn-l-ol	218.1306	C14H18O2	metab_607/metab_629 /metab_2833/metab_2870/ metab_2917/metab_5417/ metab_6230/metab_7683	metab_7683: 1.43		
Codonopilodiynoside D	398.1940	C20H30O8	metab_12357		metab_18481	metab_18481: 1.23
Codonopilodiynoside E/F/G	542.2363	C26H38O12	metab_70		metab_22437	metab_22437: 0.78;
tetradeca-4E,8E,12E-triene-10-yne-1,6,7-triol	236.1412	C14H20O3	metab_204/metab_342/ metab_6465	metab_204: 1.24; metab_342: 1.25;	metab_22708/metab_22909	

### Flavonoids and Coumarins

Flavonoids, a large group of phytometabolites, are found in almost all plants and their tissues, such as seeds, stems, flowers, leaves, roots, and fruits. Evidence has demonstrated that flavonoids have numerous health benefits for the human diet. In our metabolomics study, 21 flavonoids and 2 coumarins were detected. Among them, 14 flavonoids were identified as DEMs (Supplementary Table 3 and Table 2). Moreover, apigenin, tricin, chrysoeriol, tectoridin, and luteolin-glucopyranoside were upregulated in the stems and leaves of *C. pilosula*. Apigenin has been previously reported and discussed in detail, including its effects on diabetes, amnesia, Alzheimer’s disease, depression, insomnia, and cancer (Salehi et al., 2019). Tricin has excellent pharmacological bioactivity and numerous health benefits for human nutrition. It has been proposed as an antioxidant, antidiarrheal, antiulcer, antiallergy, and anti-inflammatory agent (Jiang et al., 2020). Recent studies found that chrysoeriol and luteolin-glucopyranoside had inhibitory activities (Kim and Jin, 2020). However, no differences were observed in quercetin, kaempferol, angelicin, and psoralen between the JY and CK groups in our metabolomics data (Supplementary Table 3). In summary, our results demonstrated that active flavonoids were more abundant in *C. pilosula* the stems and leaves than root.

**TABLE 2:** Differentially expressed metabolites (DEMs) annotated as flavonoids and coumarins.

Flavonoids and coumarins	Exact mass	Formula	ID_ [M + H] +	Fold_change	ID_[M-H]-	Fold_change
apigenin	270.0528	C15H10O5	metab_2861/metab_6124/ metab_6554/metab_12114	metab_6554: 1.54; metab_2861: 1.46; metab_12114: 1.24; metab_6124: 1.63;	metab_22669/metab_18355	metab_22669: 1.74; metab_18355: 1.43;
tricin	330.0740	C17H14O7	metab_6575	metab_6575: 2.92	metab_18654	
chrysoeriol	300.0634	C16H12O6	metab_1435/metab_6088 /metab_6577/metab_11324/ metab_11573/metab_11835	metab_11573: 1.60; metab_6088: 1.48; metab_6577: 1.45; metab_11835: 1.41; metab_11324: 1.39;	metab_18652	metab_18652: 1.39;
5-hydroxy-4′,6,7-trimethoxy flavone	328.0946	C18H16O6	metab_2889/metab_11338	metab_2889: 1.36; metab_11338: 1.33;		
5-hydroxy-4′,7-dimethoxy flavone	298.0841	C17H14O5	metab_11066	metab_11066: 1.31;		
apigenin-7-O-β-D-glucopyranoside	432.1056	C21H20O10	metab_2862/metab_5846/ metab_6122	metab_5846: 17.80; metab_6122: 1.46; metab_2862: 1.39;	metab_18212/metab_18595/metab_18433/metab_18352	metab_18433: 1.43; metab_18352: 1.42; metab_18212: 1.21;
luteolin-7-O-β-D-glucopyranoside/ luteolin-5-O-β-D-glucopyranoside/ luteolin-7-galactoside/ cynaroside	448.1005	C21H20O11	metab_319/metab_5248/ metab_5900/metab_11520/ metab_11638	metab_5248: 2.61; metab_319: 1.35; metab_11638: 1.57; metab_11520: 1.27 metab_5900: 1.20	metab_23096	metab_23096: 1.25
luteolin-7-rutinoside	594.1584	C27H30O15	metab_1295/metab_2878	metab_2878: 2.41	metab_18484/metab_17857	metab_17857: 1.49; metab_18484: 1.38;
tectoridin	462.1162	C22H22O11	metab_2830/metab_11567/metab_12033	metab_12033: 1.39; metab_2830: 1.34; metab_11567: 1.22;	metab_22721/metab_23146	metab_22721: 1.59; metab_23146: 1.35;
luteolin-7-O-D-gentibioside	610.1533	C27H30O16	metab_5651/metab_5875/ metab_11579/metab_12235/ metab_12683/metab_13057	metab_13057: 1.85; metab_5651: 1.49; metab_5875: 1.23;	metab_18332/metab_23163/ metab_16219	metab_6124: 1.63; metab_18332: 1.60; metab_23163: 1.43;
neokurarinol	470.2305	C27H34O7			metab_22512	metab_22512: 0.66;

### Lignans

Lignans are widely distributed in plants and exist in their roots, rhizomes, stems, leaves, flowers, fruits, seeds, xylem, and resins. According to studies in recent years, lignans and their derivatives from various plants possess multiple pharmacological properties, including antitumour, antioxidant, antiviral, and anti-asthmatic activities. In our study (Supplementary Table 3 and Table 3), 5 and 4 lignans were down- and upregulated in the stems and leaves of *C. pilosula*. Tangshenoside I, III, and IV were more abundant in *C. pilosula* roots. The effect of tangshenoside on hematopoietic and immunologic functions, including liver protection, has been reported by previous studies (Xin et al., 2019). In contrast, ethyl-syringin and syringaresinol accumulated more in the stems and leaves of *C. pilosula*. Previous study has shown that syringin could improve the non-specific immunity, humoral immunity, and cellular immunity of immune deficient mice (Gao et al., 2019). Overall, different types of lignans were distributed in *C. pilosula* roots, stems, and leaves.

**TABLE 3:** Differentially expressed metabolites (DEMs) annotated as lignans.

Lignans	Exact mass	Formula	ID_ [M + H] +	Fold_change	ID_[M-H]-	Fold_change
tangshenoside I	678.2371	C29H42O18			metab_18139/metab_18197	metab_18139: 0.71; metab_18197: 0.69;
tangshenoside III	726.2734	C34H46O17			metab_18357/metab_18302	metab_18357: 0.81; metab_18302: 0.80;
tangshenoside V	470.1424	C21H26O12	metab_5021	metab_5021: 0.81;		
codonoside A	824.2738	C38H48O20			metab_23286/metab_23327/ metab_18448	metab_18448: 0.8;
codonopiloneolignanin A	384.1572	C22H24O6	metab_51/metab_2555/ metab_2922/metab_12093	metab_12093: 0.70;		
ethyl-syringin	364.1733	C16H28O9			metab_23808/metab_17986	metab_17986: 1.31;
syringaresinol	418.1627	C22H26O8	metab_332/metab_2667		metab_18729	metab_18729: 1.36;
lanceolune A	206.0579	C11H10O4	metab_205		metab_17820/metab_23869	metab_17820: 1.32;
lanceolune B	220.0736	C12H12O4	metab_5296/metab_5595/ metab_13544	metab_5296: 1.21;	metab_17480/metab_18287	

### Alkaloids

Alkaloids are effective, physiologically active components in many medicinal herbs and have broad applications. Previous study showed that the total alkaloids from *C. pilosula* roots could promote the differentiation of neurite PC12 cells evoked by the nerve growth factor by strengthening the upstream phase of the MAPK-dependent signalling pathway (Liu et al., 2003). In this study, 26 alkaloids were annotated from *C. pilosula*, 11 of which were identified as DEMs in the comparison of the JY and CK groups (Supplementary Table 3 and Table 4). The relative content of codotubulosine A and codonopsinine in the roots was lower than that in the leaves and stems. However, the relative contents of codonopsinol A and C, codonopiloside A, radicamine A, and codonopsine 2 in roots were higher than that in the leaves and stems. A previous study suggested that pyrrolidine alkaloid radicamine A, a novel drug for diabetes treatment, could significantly inhibit glucose absorption in the small intestine (Liu et al., 2010). Interestingly, some alkaloids were obviously more prevalent in leaves and stems, whereas the content of some bioactive alkaloids were significantly enriched in roots.

**TABLE 4:** Differentially expressed metabolites (DEMs) annotated as alkaloids.

Alkaloids	Exact mass	Formula	ID_ [M + H] +	Fold_change	ID_[M-H]-	Fold_change
codotubulosine A	268.1549	C14H22NO4 +	metab_4052	metab_4052: 1.48;	metab_23809	metab_23809: 1.92;
codonopsinine	237.1365	C13H19NO3	metab_4777/metab_12434/ metab_12514/metab_13702	metab_12514: 2.10; metab_12434: 1.64;		
adenosine	267.0968	C10H13N5O4			metab_23895	metab_23895: 1.59;
hypoxanthine	136.0385	C5H4N4O	metab_88/metab_14595	metab_88: 1.305;	metab_24200/metab_24013	metab_24200: 1.23; metab_24013: 1.22;
Codonopsinol C	239.1158	C12H17NO4	metab_758/metab_833/ metab_1045/metab_4282/ metab_12140/metab_14615	metab_14615: 0.73;	metab_23768/metab_18030/ metab_22929	metab_23768: 0.78;
Codonopiloside A	415.1842	C19H29NO9	metab_1090		metab_16058/metab_22995	metab_16058: 0.82; metab_22995: 0.74;
Codonopsinol A	269.1263	C13H19NO5	metab_975/metab_1104/ metab_12456/metab_13025/ metab_13520/metab_13754		metab_18056/metab_17874/ metab_17601/metab_18010	metab_17874: 0.80; metab_18010: 0.78; metab_18056: 0.68;
radicamine A	255.1107	C12H17NO5	metab_224/metab_1004/ metab_1315/metab_1346/ metab_2965/metab_4086/ metab_13767/metab_14567/ metab_14573/metab_15411	metab_1346: 0.82; metab_1315: 0.78;	metab_23949/metab_17650	
6-methoxy-4-formyl quinoline/ 6-methoxyquinoline-4-carbaldehyde	187.0633	C11H9NO2	metab_212/metab_1390/ metab_5634/metab_11778/ metab_12152	metab_12152: 1.30; metab_1390: 1.22;		
N-9-formyl harman	210.0793	C13H10N2O	metab_10361		metab_23170	metab_23170: 0.83;
Codonopsine 2	267.1470	C14H21NO4	metab_40/metab_13066		metab_23644/metab_16335	metab_16335: 0.56;

### Terpenoids

Terpenoids are the largest and most diverse group of phytometabolites among plant-based compounds. Traditionally, terpenoids produced by plants have been applied in the food, health supplement, pharmaceutical, and chemical industries, and more recently in biofuel products (Tholl, 2015). Despite reports that 48 terpenoids and their glycosides were found in *Codonopsis*, only 14 of them were detected in our metabolomics analysis. Moreover, the content of eight terpenoids, including atractylenolide III, oleanolic acid, and echinocystic acid, was higher in the leaves and stems than in the roots (Supplementary Table 3 and Table 5). The pharmacological properties of atractylenolide III, a major active constituent of *Atractylodes* rhizomes, include anti-inflammatory, gastroprotective, and neuroprotective effects (Wang et al., 2019). Oleanolic acid and its derivatives have been widely used for treating hepatopathy and have been suggested to have antiosteoporosis, antidiabetic, antibacterial, anticancer, and haemolytic effects. As a natural compound from *Gleditsia sinensis*, echinocystic acid (EA) exhibits anti-inflammatory, antioxidant, and analgesic activities. Some studies have shown that EA has anticancer properties, inducing apoptosis in tumour cells; however, in the nervous system, EA promoted the proliferation and growth of nerve protrusion in the hippocampal regions of elderly mice (Chen B. et al., 2020). Our analysis identified that at least eight terpenoids significantly accumulated in leaves and stems compared to roots.

**TABLE 5:** Differentially expressed metabolites (DEMs) annotated as terpenoids.

Terpenoids	Exact mass	Formula	ID_ [M + H] +	Fold_change	ID_[M-H]-	Fold_change
Atractylenolide III	248.1412	C15H20O3	metab_595/metab_10027	metab_10027: 1.27;	metab_21983	
oleanolic acid	456.3603	C30H48O3			metab_20401/metab_19996/ metab_20753/metab_19268/ metab_21359/metab_20851/ metab_21055	metab_20401: 1.47; metab_19268: 1.27; metab_19996: 1.23; metab_20851: 1.22;
echinocystic acid	472.3553	C30H48O4	metab_409/metab_452/ metab_7630/metab_8608/metab_8996	metab_8608: 1.69; metab_7630: 1.51;	metab_21966/metab_20646/ metab_21751/metab_16471	metab_20646: 1.36; metab_21966: 1.36; metab_21751: 1.27;
8β-hydroxyasterolid/ atractylenolide	248.1412	C15H20O3	metab_595/metab_10027	metab_10027: 1.27;	metab_21983	
albigenic acid	472.3553	C30H48O4	metab_409/metab_452 /metab_7630/metab_8608/ metab_8996	metab_8608: 1.69; metab_7630: 1.51;	metab_21966/metab_20646/ metab_21751/metab_16471	metab_20646: 1.36; metab_21966: 1.36; metab_21751: 1.27;
rubiprasin	516.3814	C32H52O5	metab_2231	metab_2231: 1.3124;	metab_20007/metab_20333/ metab_20727/metab_16436	metab_16436: 1.66; metab_20333: 1.35;
hopane-6α,22-diol	444.3967	C30H52O2			metab_21195/metab_16586	metab_21195: 1.53;

### Steroids

Steroids have been generally recognised as the hormones of higher vertebrates. There is compelling evidence, particularly from recent studies, that steroids are also essential for normal plant growth and development. Recent study has shown that plant sterols and their derivatives are inhibitors of intestinal cholesterol absorption and agents for lowering total plasma and low-density lipoprotein (LDL) cholesterol levels (Zhabinskii et al., 2015). Steroids have been known as a source of novel leads in the development of therapeutics for cancer treatment. In this study, 9 steroids and their glycosides were detected in *C. pilosula* using metabolomics analysis (Supplementary Table 3 and Table 6). Among them, the content of five steroids, including Δ7-stigmasterol and Δ5,22-stigmasterol, were higher in leaves and stems than in roots. The therapeutic potential of stigmasterol, a natural steroid alcohol with established immune-modulatory properties, was previously assessed on allergic cutaneous responses. In addition, a previous study confirmed the binding potential of Δ7-stigmasterol from sesame oil with cyclooxygenase-2, having a major role in anti-inflammatory activity (Afroz et al., 2019). In general, steroids were more prevalent in *C. pilosula* stems and leaves.

**TABLE 6:** Differentially expressed metabolites (DEMs) annotated as steroids.

Steroids	Exact mass	Formula	ID_ [M + H] +	Fold_change	ID_[M-H]-	Fold_change
Δ7-stigmasterol	410.3548	C29H46O	metab_1986/metab_2080	metab_1986: 1.20;	metab_20790/metab_20411/ metab_16056/metab_21084	metab_20411: 1.93; metab_16056: 1.43; metab_21084: 1.24;
Δ7-stigmasteryl glucoside	572.4076	C35H56O6			metab_20693/metab_22287/ metab_20288	metab_20693: 0.78;
Δ7-stigmasta-7-ene-3-one	408.3392	C29H44O	metab_7751/metab_9051	metab_9051: 1.25; metab_7751: 1.24;	metab_20793/metab_20111/ metab_21241/metab_20355/ metab_20241	metab_20793: 2.03; metab_20355: 1.90; metab_20241: 1.64; metab_20111: 1.23;
Δ5,22-stigmasterol	456.3603	C30H48O3	metab_7535/metab_7759/ metab_8715	metab_7759: 1.29;	metab_20401/metab_19996/ metab_20753/metab_19268/ metab_21359/metab_20851/ metab_21055	metab_20401: 1.47; metab_19268: 1.27; metab_19996: 1.23; metab_20851: 1.22;
stigmast-7,22-dien-3-ol	410.3548	C29H46O	metab_1986/metab_2080	metab_1986: 1.20;	metab_20790/metab_20411/ metab_16056/metab_21084	metab_20411: 1.93; metab_16056: 1.43; metab_21084: 1.24;

### Organic Acids

Multiple pharmacological studies have demonstrated the beneficial potential of organic acids for human health. In recent years, organic acids and their derivatives have also been used for plant protection against pathogens and pests, focusing on microbiological processes for the production of these high-quality microbial metabolites from available, inexpensive, and renewable substrates (Izquierdovega et al., 2020). Almost all organic acids recorded in previous studies of *C. pilosula* were identified in our metabolomics analysis (Supplementary Table 3 and Table 7). Among them, the relative content of seven organic acids, including lauric acid, caffeic acid, and chlorogenic acid, was higher in stems and leave than in roots, while the relative content of syringic acid was lower in stems and leaves than in root. In recent study, lauric acid has been proven to contribute multiple benefits to human health. Lauric acid lowered blood pressure and oxidative stress in normotensive and hypertensive rats and diminished the inflammatory response in THP-1 cells induced by *Propionibacterium acnes* (Yong et al., 2020). Caffeic acid and related phenylpropanoic acids are ubiquitous natural products of the shikimic acid pathway (Habtemariam, 2017). Due to the presence of diorthohydroxyl aromatic (catecholic) moiety, caffeic acid possesses numerous pharmacological effects, ranging from anti-inflammatory to anti-cancer effects. Chlorogenic acid (5-O-caffeoylquinic acid), a phenolic compound from the hydroxycinnamic acid family, displays different health-promoting properties, including antioxidant, anti-inflammatory, antilipidemic, antidiabetic, and antihypertensive activities, most of which are related to the treatment of metabolic syndrome (Jesús et al., 2017). Syringic acid possesses antioxidant, antimicrobial, anti-inflammatory, and anti-endotoxic properties (Liu et al., 2020). Taken together, organic acids were much more abundant in the leaves and stems than in the roots of *C. pilosula*.

**TABLE 7:** Differentially expressed metabolites (DEMs) annotated as organic acids.

Organic acids	Exact mass	Formula	ID_ [M + H] +	Fold_change	ID_[M-H]-	Fold_change
lauric acid	200.1776	C12H24O2			metab_21476/metab_22226/ metab_19308	metab_21476: 1.35;
2,4-non-adlenic acid	166.0993	C10H14O2	metab_1230/metab_1478/ metab_6441	metab_1230: 1.25;	metab_22740/metab_22350	metab_22740: 1.5;
caffeic acid	180.0423	C9H8O4	metab_3821/metab_5102/ metab_6148/metab_10908/ metab_12643/metab_16008	metab_10908: 1.33; metab_12643: 1.22; metab_6148: 1.22;	metab_19396	
chlorogenic acid/ neochlorogenic acid/ 3-O-Caffeoylquinic acid	354.0951	C16H18O9	metab_22/metab_5893	metab_5893: 1.81;	metab_23996	
8-O-4′ diferulic acid	386.1001	C20H18O8			metab_23185	metab_23185: 1.33;
syringic acid	198.0528	C9H10O5	metab_214/metab_3200/ metab_11451/metab_14616/ metab_15076	metab_14616: 0.76; metab_11451: 0.73;		

### Detection of Lobetyolin, Syringin and Atractylenolide III

**FIGURE 3:**
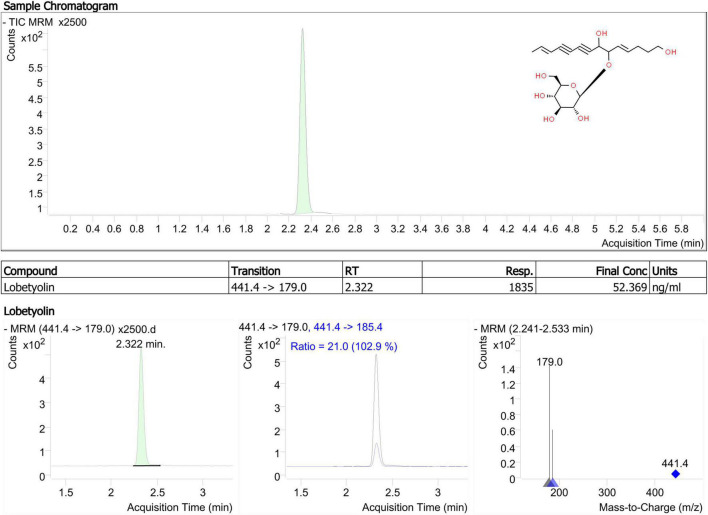
Product ion mass spectra of [M + COOH]^–^ ion of lobetyolin and chromatography peak of a representative JY sample (retention time 2.322 min).

Lobetyolin, a major bioactive constituent, was used as a marker for TLC identification of Radix Codonopsis in the China Pharmacopoeia Committee (2020). Based on a previous study, it is more common to increase sensitivity for lobetyolin detection by applying electrospray ionisation (ESI) in negative mode (Zhang et al., 2013). As shown in Figure 3, lobetyolin was identified from the standard reference and by analysing the ion fragmentation patterns. Here, the lobetyolin content in samples from JY groups were 0.142 ± 0.011 mg/g (0.0142%). There results were similar to those in a previous study (Chen M. et al., 2020). Despite that the general content in fresh leaves (0.039%), stems (0.027%), and roots (0.021%) of *C. pilosula* was not high, a previous study found that lobetyolin in the roots had a similar or slightly higher content (0.01–0.07%) due to the different detection method (Chen M. et al., 2020). As previously (see section “Polyacetylenes, Polyenes, and Their Glycosides”) mentioned, similar results were reported in our metabolomics data. Our results suggested that *C. pilosula* stems and leaves also contained lobetyolin and have a potential healthcare value in immunity enhancement.

Syringin exhibits multiple pharmacological properties, including anti-angiogenic and anti-inflammatory effects. Atractylenolide III, a sesquiterpene lactone, is the major antioxidant from Atractylodes rhizome. They were also quality markers for *C. pilosula* root (Bai Y. E. et al., 2020). As shown in HPLC analysis (Supplementary Figure 3), the content of syringin in samples from JY and CK groups were 0.329 ± 0.008 mg/g and 0.191 ± 0.014 mg/g, respectively. In the metabonomics data, syringin (metab_5680 and metab_5632) was not DEMs, however, the content of ethyl-syringin (metab_17986) was higher in leaves and stems than in roots. Moreover, there was no difference of the content of atractylenolide III between the two groups (0.018 ± 0.004 mg/g in JY group and 0.024 ± 0.005 mg/g in CK group). Here, syringin and atractylenolide III were also identified in the dried aerial parts of *C. pilosula*, indicating a potential healthcare value. Similar findings were reported in a previous study of *C. nervosa* aerial parts (Aga et al., 2012).

### Antioxidant Activity

In FRAP assay, the ferric reducing capacity of *C. pilosula* extracts was determined with reference to the reaction signal given by a Fe^2+^ solution. The free radical scavenging capacity of *C. pilosula* extracts was detected by the ABTS radical cation decolorization assay. In the FRAP assay, the aerial part (0.49 ± 0.01 mM) extracts possessed higher antioxidant capacity than the roots (0.42 ± 0.02 mM) of *C. pilosula*. This trend for the free radical scavenging capacity of the aerial part (0.68 ± 0.05 mM, Trolox equivalent antioxidant capacity) and root (0.42 ± 0.03 mM) extracts was even more clearly. Our results suggested that the extracts of *C. pilosula* aerial parts showed strong antioxidant properties.

## Conclusion

In this study, we analysed and compared chemical constituents derived from the stems and leaves, the agricultural waste materials, and roots, the medicinal materials, of *C. pilosula*. Based on the UPLC-MS data, metabolites were classified and identified by our integrated bioinformatics pipeline. In total, 1508 metabolites were identified; 463 were identified as DEMs and grouped into classes of carboxylic acids and derivatives, steroids, organic oxygen compounds, fatty acyls, prenol lipids, flavonoids, and others. Metabolic profiling of *C. pilosula* tissues implied that polyacetylenes, polyenes, flavonoids, some alkaloids, steroids, terpenoids, and organic acids were accumulated in leaves and stems, whereas lignans and some alkaloids were enriched in the roots. In addition, lobetyolin, syringin and atractylenolide III were also detected in *C. pilosula* stems and leaves. The extracts of *C. pilosula* aerial parts showed stronger antioxidant properties than roots. Our results suggested that *C. pilosula* stems and leaves were also rich in multiple pharmaceutical metabolites and might have a very high value in therapeutic healthcare.

## Data Availability Statement

The original contributions presented in the study are included in the article/Supplementary Material, further inquiries can be directed to the corresponding author/s.

## Author Contributions

XZ, JL, XC, and SG discussed and plan the work and wrote the manuscript. XZ, JL, and XL conducted the experiments. XZ and JC carried out the data analysis, created the figures, and drafted the initial manuscript. All authors commented, made corrections, and approved the submitted version.

## Conflict of Interest

The authors declare that the research was conducted in the absence of any commercial or financial relationships that could be construed as a potential conflict of interest.

## Publisher’s Note

All claims expressed in this article are solely those of the authors and do not necessarily represent those of their affiliated organizations, or those of the publisher, the editors and the reviewers. Any product that may be evaluated in this article, or claim that may be made by its manufacturer, is not guaranteed or endorsed by the publisher.

## Footnotes

1
https://metlin.scripps.edu/

2
http://www.hmdb.ca/

3
https://www.metaboanalyst.ca/

## Supplementary Material

Supplementary Figure 1 Representative UPLC-MS typical base peak intensity chromatograms of QC samples from positive and negative ion modes.

Supplementary Figure 2 The standard curve of lobetyolin.

Supplementary Figure 3 HPLC analyses of syringin and atractylenolide III. **(A)** syringin; **(B)** atractylenolide III; **(C)** sample from CK group; **(D)** sample from JY group.
