# Supplementary figures and images for: Untargeted Metabolomics Reveals Multiple Phytometabolites in the Agricultural Waste Materials and Medicinal Materials of Codonopsis pilosula
Source: Front Plant Sci. 2022 Jan 10;12:814011. doi: 10.3389/fpls.2021.814011 (PMC8784785; doi:10.3389/fpls.2021.814011)

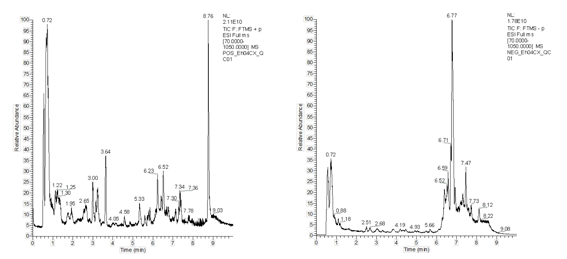

Supplement: Supplementary Figure 1 — Representative UPLC-MS typical base peak intensity chromatograms of QC samples from positive and negative ion modes. [file Image_1.TIF]

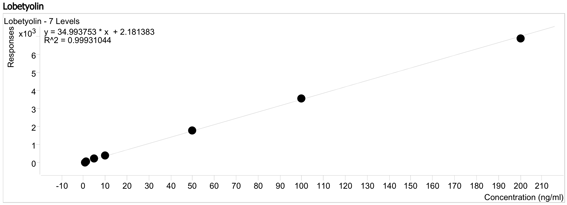

Supplement: Supplementary Figure 2 — The standard curve of lobetyolin. [file Image_2.TIF]

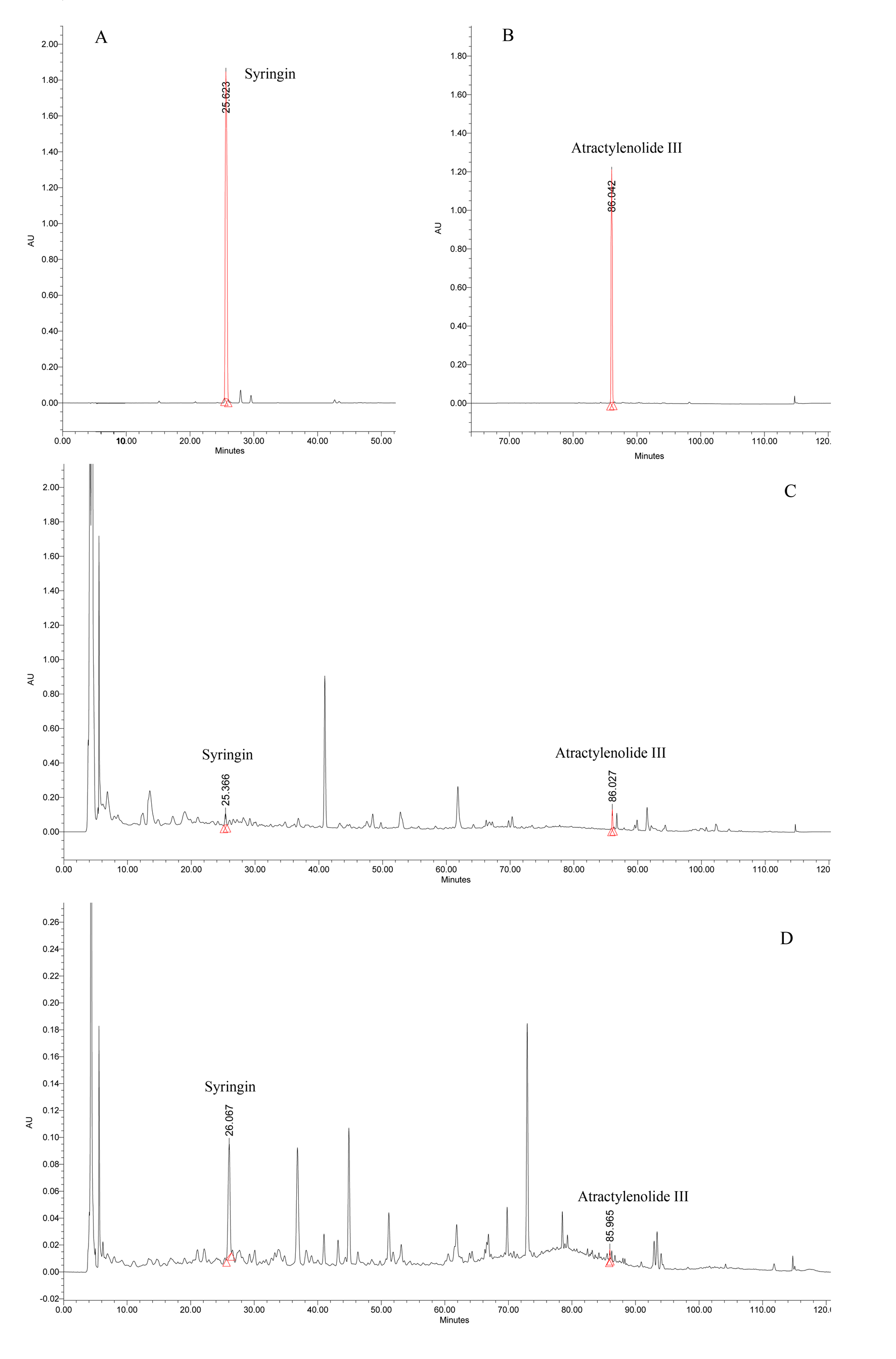

Supplement: Supplementary Figure 3 — HPLC analyses of syringin and atractylenolide III. (A) syringin; (B) atractylenolide III; (C) sample from CK group; (D) sample from JY group. [file Image_3.TIF]
